# Efficacy of a single intra-articular injection of mesenchymal stem cells for knee osteoarthritis: a dose-focused meta-analysis of randomized controlled trials

**DOI:** 10.1186/s13018-025-06190-4

**Published:** 2025-08-30

**Authors:** Rizki Rahmadian, Zikril Ariliusra, Kevin Rizki Agung Kusuma, Hirowati Ali, Benni Raymond, Riki Meksiko

**Affiliations:** 1 Orthopaedic and Traumatology Division, Department of Surgery, Faculty of Medicine, M. Djamil General Hospital, Universitas Andalas, Padang, Indonesia; 2 Tissue Bank and Cell Department, M. Djamil General Hospital, Padang, Indonesia; 3 Tarakan General Hospital, Central Jakarta, Jakarta, Indonesia; 4 Lanud Eltary Hospital, Kupang, East Nusa Tenggara, Indonesia; 5 Department of Biochemistry, Faculty of Medicine, Universitas Andalas, Padang, Indonesia; 6 Plastic Surgery Division, Department of Surgery, Faculty of Medicine, M. Djamil General Hospital, Universitas Andalas, Padang, Indonesia

**Keywords:** Intra-articular injection, Mesenchymal stem cell, Knee osteoarthritis

## Abstract

**Background:**

Intra-articular injection of mesenchymal stem cells (MSCs) has emerged as a promising therapy for knee osteoarthritis (OA). However, uncertainty remains regarding the optimal cell dose for maximizing clinical benefit. This systematic review and meta-analysis aimed to evaluate the efficacy of MSC injections for knee OA and investigate the relationship between cell dose and treatment outcome.

**Methods:**

A comprehensive literature search was conducted in PubMed and Scopus for randomized controlled trials (RCTs) published between 2015 and 2025. Eligible studies were RCTs that used intra-articular MSC injections for knee OA, and WOMAC scores were reported at baseline and 12-month follow-up. Standardized mean differences (SMDs) were pooled using a random-effects model. Subgroup analysis and meta-regression were performed to evaluate the effect of MSC dose on treatment outcomes.

**Results:**

Eight treatment arms from six RCTs involving 300 patients were included. The pooled SMD in the WOMAC score at 12 months was –1.35 (95% CI: –1.97 to –0.74), indicating a moderate to large treatment effect. MSC doses of ≤ 25 million cells were associated with statistically significant improvement, while higher doses did not demonstrate additional benefit. Meta-regression confirmed no significant dose–response relationship. Heterogeneity was moderate (I2 = 49.8%).

**Conclusion:**

Intra-articular MSC therapy significantly improves clinical outcomes in knee OA at 12 months, with lower doses (≤ 25 million cells) appearing to be both effective and potentially more efficient. These findings support dose optimization as a critical consideration in advancing MSC therapy.

## Introduction

Knee osteoarthritis (OA) is a highly prevalent degenerative joint disease and a leading cause of pain and disability worldwide, affecting approximately 654 million individuals aged 40 and above[1]. The growing prevalence, driven by aging populations and rising obesity rates, results in a significant economic burden, with annual OA-related healthcare and productivity costs estimated at over $185 billion in the United States alone [2], and up to €26.9 million in workforce losses due to sick leave in the Netherlands[3]. These figures underscore the urgent need for effective, disease-modifying therapies to alleviate both the clinical and financial impact of knee OA.

The limited regenerative capacity of articular cartilage and the shortcomings of current treatment strategies have led to increased interest in regenerative approaches, particularly mesenchymal stem cells (MSCs) for knee osteoarthritis (OA)[4, 5]. MSCs possess multipotent differentiation capabilities, self-renewal potential, and immunomodulatory effects, making them a promising therapeutic candidate for cartilage repair[6, 7]. Intra-articular injection, in particular, is considered a safer and simpler method, avoiding complications related to surgical implantation[2]. Evidence from randomized clinical trials and meta-analyses suggests that MSC injections may reduce pain and improve joint function, with some studies reporting increased cartilage volume and regeneration of hyaline-like tissue as seen on MRI or arthroscopy[6, 8]. While results are encouraging, optimal dosing, long-term durability, and mechanisms of action are still under investigation[9, 10].

Several factors, including glucose availability, oxygen levels, and the presence of growth factors influence the survival of mesenchymal stem cells (MSCs)[11, 12]. Like other cell types, MSCs rely on adequate nutrient intake and cellular respiration, both of which depend on the surrounding vascularization[13–16]. However, the intra-articular space lacks direct vascularization, relying instead on the diffusion of nutrients and oxygen from the surrounding synovial membrane[17]. This creates a relatively hypoxic and nutrient-limited microenvironment for injected MSCs, which may compromise their survival and engraftment. As such, administering an excessively high dose of MSCs may lead to increased cell death due to limited nutrient availability and heightened resource competition. These considerations highlight the importance of determining an optimal dosing strategy that balances therapeutic efficacy with cell survival in the joint environment.

## Method

### Eligibility criteria

Studies were selected based on predefined inclusion and exclusion criteria to ensure methodological rigor and relevance to the research question.

Inclusion criteria were as follows:

- Randomized controlled trials (RCTs)
- At least a patient-blinded design
- Reported Western Ontario and McMaster Universities Osteoarthritis Index (WOMAC) scores at both baseline and 12-month follow-up
- Investigated the use of intra-articular injection of mesenchymal stem cells (MSCs) for knee osteoarthritis
- Did not include any additional invasive treatments

Exclusion criteria included:

- Non-randomized trials or open-label studies
- Studies with high risk in overall RoB 2 assessment
- Studies that administered MSCs via multiple injections or in combination with surgery therapies
- The MSC dose is not specified
- Studies that did not report WOMAC outcomes at the specified follow-up time

### Information sources

We searched two electronic databases: Scopus and PubMed. The searches included studies published between January 2015 and March 2025, and were limited to English-language articles. The last search was conducted in March 2025. Additional articles were identified by manually screening the reference lists of included studies and relevant systematic reviews.

### Search strategy

A comprehensive literature search was conducted in Scopus and PubMed to identify randomized controlled trials (RCTs) evaluating the efficacy of intra-articular mesenchymal stem cell (MSC) injections for knee osteoarthritis. The search included studies published between January 2015 and March 2025 and was limited to articles published in English. This systematic review protocol was registered with PROSPERO (International Prospective Register of Systematic Reviews) under the ID CRD420251107915.

In Scopus, the following search string was used:

TITLE-ABS-KEY("knee osteoarthritis" OR "knee OA") AND TITLE-ABS-KEY("stem cell" OR "mesenchymal stem cell" OR "MSC" OR "adipose-derived stem cell" OR "bone marrow-derived stem cell" OR "Wharton's jelly stem cell") AND TITLE-ABS-KEY("intra-articular injection" OR "intraarticular injection") AND TITLE-ABS-KEY("randomized controlled trial" OR "randomised controlled trial" OR "RCT" OR "randomized clinical trial") AND LIMIT-TO(DOCTYPE,"ar") AND LIMIT-TO(SUBJAREA, "MEDI") AND LIMIT-TO(SRCTYPE, "j") AND LIMIT-TO(LANGUAGE, "English") AND PUBYEAR > 2014 AND PUBYEAR < 2026.

In PubMed, the following search string was used:

("knee osteoarthritis" OR "knee OA") AND ("stem cell" OR "mesenchymal stem cell" OR "MSC" OR "adipose-derived stem cell" OR "bone marrow-derived stem cell" OR "Wharton's jelly stem cell") AND ("intra-articular injection" OR "intraarticular injection") AND (humans[MeSH Terms]).

The reference lists of eligible studies and relevant reviews were also screened manually to identify additional studies not captured by the database search.

### Study selection

The study selection process was carried out independently by three reviewers. All identified records were imported into Rayyan for screening. Duplicate entries were removed using Rayyan's automated duplicate detection tool. Titles and abstracts were then screened for eligibility within Rayyan. Studies that met the initial criteria were retrieved in full text and evaluated one by one by the reviewers. Disagreements during any stage of the selection process were resolved through discussion to reach consensus.

### Data extraction process

Data extraction was carried out independently by three reviewers using a standardized data extraction form. The following information was collected from each included study:

- Study details (author, year, country)
- Sample size
- Source and type of mesenchymal stem cells (MSCs)
- MSC dose
- Comparator intervention
- Follow-up duration
- Outcome measures

Any discrepancies during data extraction were resolved through discussion among the reviewers. If necessary, a consensus was reached with the involvement of all three reviewers.

### Risk of bias assessment

The risk of bias for each included randomized controlled trial was assessed using the Cochrane Risk of Bias 2 (RoB 2) tool. The assessment was conducted independently by the three reviewers (ZA, KRAK, RM). Any discrepancies were discussed until consensus was achieved.

### Data synthesis

Data synthesis was performed using R software (version 4.5.0), employing the meta and metafor packages. For continuous outcomes, such as the WOMAC total score, standardized mean differences (SMDs) and 95% confidence intervals (CIs) were calculated. A random-effects model was applied to account for between-study variability. Heterogeneity was assessed using the I2 statistic, with values above 50% interpreted as moderate to high heterogeneity. A forest plot was generated to illustrate effect sizes and confidence intervals across studies.

### Meta-regression analysis

Meta-regression was conducted using the metafor package in R to examine whether the MSC dose influenced the treatment effect. Doses were categorized into two groups: ≤ 25 million and > 25 million cells. A mixed-effects meta-regression model was employed, and the Knapp-Hartung adjustment was applied to enhance the accuracy of standard error estimation and p-values, particularly given the limited number of included studies. Regression coefficients, confidence intervals, and p-values were reported to assess the strength of association between MSC dose and treatment efficacy.

## Result

### Study selection

A systematic literature search identified 59 unique records after 26 duplicates were removed. Automation tools or other filters excluded no records. All 59 records were screened based on titles and abstracts, resulting in 30 articles selected for full-text review. No reports were unavailable or excluded due to retrieval issues.

**Fig. 1:**
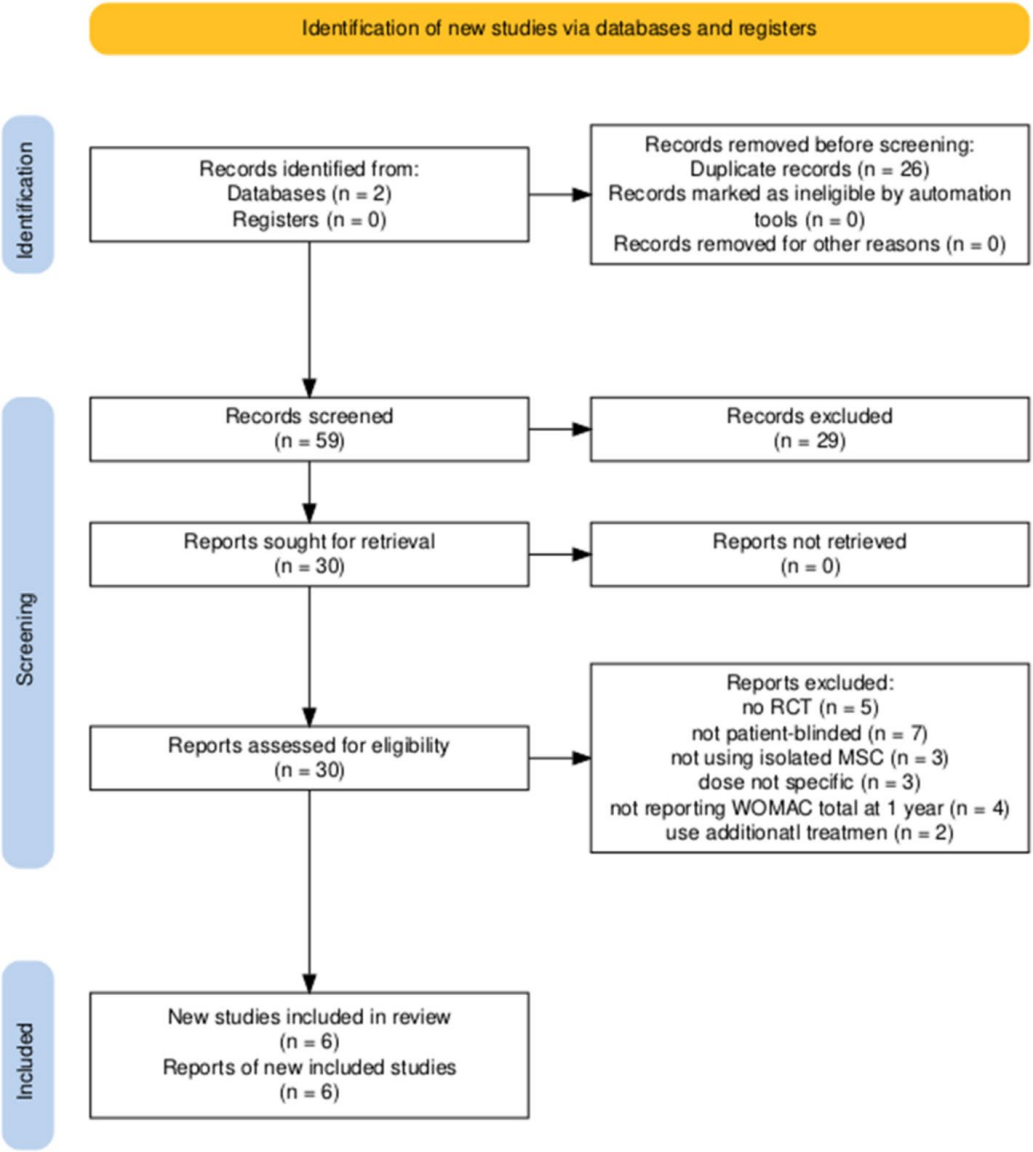
PRISMA flow diagram of study selection [18]

Following full-text assessment, 24 articles were excluded for the following reasons: not being randomized controlled trials (*n* = 5), lacking patient-blind design (*n* = 7), not using isolated mesenchymal stem cells (MSC) (*n* = 3), unspecified MSC dosing (*n* = 3), absence of WOMAC total score reporting at 12 months (*n* = 4), or combining MSCs with additional interventions (*n* = 2). Ultimately, six studies were deemed eligible and included in the systematic review and meta-analysis (Fig. 1).

A total of six randomized controlled trials were included in this review, as summarized in Table 1, and the risk of bias assessment for the included randomized controlled trials is summarized in Table 1. The studies were conducted across diverse geographical regions, including Iran, Spain, South Korea, India, Taiwan, Chile, and Colombia. Most studies used allogeneic MSCs derived from adipose tissue, bone marrow, or umbilical cord sources. The administered doses ranged from 16 × 10^6^ to 100 × 10^6^ cells, with all studies utilizing a single intra-articular injection protocol. Follow-up durations ranged from 48 weeks to 12 months, and all studies reported changes in WOMAC scores, with several also evaluating VAS, KOOS, SF-36, and imaging outcomes such as MRI or biomarkers.

**Table 1:** Summary of included studies

Study (Author, Year)	Country	Sample Size	MSC Source vs Control	Dose (cells)	Follow-up Duration	Outcomes Measured
[19]	Iran	40(20 MSC)	AD (allogeneic) vs saline	100 $\times$ 10^6^	12 months	WOMAC, VAS, KOOS, SF-36, MRI, biomarkers
[20]	Spain	30 (15 MSC)	BM (allogeneic) vs HA	40 $\times$ 10^6^	12 months	WOMAC, VAS, Lequesne Index, SF-12, MRI (T2 mapping)
[21]	South Korea	24 (12 MSC)	BM (allogeneic) vs saline	100 $\times$ 10^6^	12 months	WOMAC, VAS, KOOS, MRI (T2 mapping), biomarkers
[22]	India	146 (73 MSC)	BM (allogeneic) vs HA	25 $\times$ 10^6^	12 months	WOMAC, VAS, MRI (T2 mapping), CTX-II, IL-10
[23]	Taiwan	57 (49 MSC arms)	AD (allogeneic) vs HA	16, 32, 64 $\times$ 10^6^	48 weeks	WOMAC, VAS, KSCRS
[24]	Chile	27 (9 MSC single-dose, eight control)	UC (allogeneic) vs placebo	20 $\times$ 10^6^	12 months	WOMAC, VAS, SF-36, OMERACT-OARSI, MRI (WORMS)

A total of six randomized controlled trials were included in the meta-analysis, contributing eight independent MSC treatment arms. Chen et al. [23] included three parallel arms based on different MSC doses (16 million, 32 million, and 64 million cells), each analyzed as a separate comparison against a control group. Across these arms, the standardized mean differences (SMDs) in WOMAC scores at 12-month follow-up ranged from –0.39 to –2.99. The largest effect was observed in the 32 million cell group of Chen et al. [23] (SMD = –2.985, 95% CI: –4.578 to –1.392), while Lee et al. (2019) reported the most minor and statistically non-significant effect (SMD = –0.391, 95% CI: –1.217 to 0.435) (Table 2). Seven of the eight comparisons showed statistically significant improvement in WOMAC scores, supporting the efficacy of intra-articular MSC injections for knee osteoarthritis (Table 3).

**Table 2:** RoB 2 score for included studies

Study	Randomization	Deviations	Missing Data	Outcome Measurement	Reporting	Overall RoB	Reason for'Some concerns'
[20]	Some concerns	Low risk	Low risk	Low risk	Low risk	Some concerns	Sequence generation & concealment are not described
[19]	Low risk	Low risk	Low risk	Low risk	Low risk	Low risk	—
[24]	Some concern s	Low risk	Low risk	Low risk	Low risk	Some concerns	Random sequence generation not reported
[21]	Low risk	Low risk	Low risk	Low risk	Low risk	Low risk	—
[22]	Low risk	Low risk	Low risk	Low risk	Low risk	Low risk	—
[23]	Low risk	Some concerns	Low risk	Low risk	Low risk	Some concerns	The study was single-blind (participant-blinded)

**Table 3:** Standardized mean differences (SMD) and 95% confidence intervals of WOMAC total score at 12 months for included studies

Study	SMD	SE SMD	95% CI Lower	95% CI Upper
Chen 2021 (16 M)	−1.584	0.705	−2.967	−0.202
Chen 2021 (32 M)	−2.985	0.813	−4.578	−1.392
Chen 2021 (64 M)	−1.954	0.741	−3.407	−0.501
Gupta 2023	−1.266	0.181	−1.621	−0.910
Lee 2019	−0.391	0.421	−1.217	0.435
Matas 2018	−0.701	0.501	−1.682	0.280
Sadri 2023	−1.657	0.386	−2.414	−0.899
Vega 2015	−1.434	0.409	−2.236	−0.631

*SMD* standardized mean difference, *SE* standard error, *CI* confidence interval. Negative values indicate improvement in WOMAC scores in favor of MSC over control

## Forest plot

**Fig. 2:**
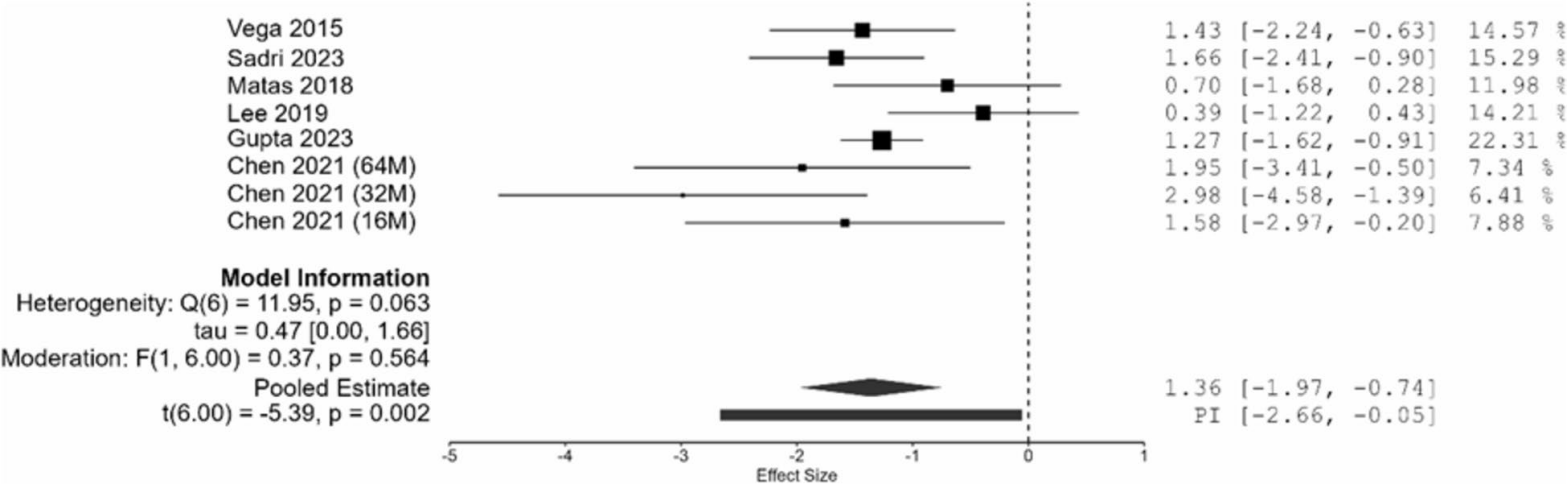
Forest plot of included studies

The meta-analysis of eight randomized controlled trials demonstrated a significant improvement in WOMAC scores at 12-month follow-up among patients receiving intra-articular MSC injections compared to controls (SMD = –1.35, 95% CI: –1.97 to –0.74, *p* = 0.002), as shown in Fig. 2. Heterogeneity across studies was moderate (Q(6) = 11.95, *p* = 0.063; I2 = 49.8%; τ2 = 0.47). The results suggest a moderate-to-large beneficial effect of MSC therapy for knee osteoarthritis.

Meta-regression analysis indicated that MSC injections with doses of ≤ 25 million cells significantly improved WOMAC scores at 12 months (SMD = –1.165, 95% CI: –2.110 to –0.220, *p* = 0.023), as shown in Table 4. In contrast, doses > 25 million cells did not result in a statistically significant difference in effect compared to lower doses (β = –0.310, 95% CI: –1.552 to 0.932, *p* = 0.564).

**Table 4:** Meta-regression summary

						95% CI	
	Estimate	Standard Error	t	df	p	Lower	Upper
Intercept	−1.165	0.386	−3.017	6.000	0.023	−2.110	−0.220
> 25 million	−0.310	0.508	−0.610	6.000	0.564	−1.552	0.932

Fixed effect tested using Knapp and Hartung adjustment

## Discussion

This meta-analysis demonstrates that intra-articular mesenchymal stem cell (MSC) therapy significantly improves symptoms in patients with knee osteoarthritis, as reflected by reduced WOMAC scores at 12 months post-injection. Across six randomized controlled trials contributing eight independent treatment arms, the pooled standardized mean difference (SMD) was −1.35 (95% CI: −1.97 to –0.74), indicating a moderate to large treatment effect. Seven MSC arms showed statistically significant improvements compared to controls, suggesting consistent clinical benefit across different MSC sources and study populations. Further analysis revealed that MSC doses of 25 million cells or less were associated with a statistically significant improvement, while higher doses did not confer a significant additional benefit. This finding highlights the potential effectiveness of lower MSC doses and raises important considerations for dose optimization in clinical practice.

Numerous prior studies and reviews have investigated the effectiveness of intra-articular MSC injections for knee osteoarthritis, and the current results substantially align with these findings. Our meta-analysis identified moderate-to-large impact sizes that correspond with previous studies, indicating that MSCs may surpass traditional intra-articular treatments, including hyaluronic acid and corticosteroids, in enhancing pain relief and functional outcomes. A meta-analysis conducted by Yubo et al. [25] revealed that MSC therapy markedly enhanced VAS, WOMAC, and several other clinical scores, with enduring benefits observed over 12 to 24 months and no notable safety issues, thereby underscoring the therapeutic potential of MSCs [25]. Cui et al. [26] reviewed 18 clinical studies, revealing substantial enhancements in pain and function post-MSC therapy,nevertheless, they observed an absence of a definitive dose–response association and greater effect sizes in non-randomized trials [26]. They noted enhanced efficacy when MSCs were utilized alongside activation agents and in the early stages of OA. Shariatzadeh et al. [27] emphasize that significant variety exists in MSC trial designs, encompassing cell sources, expansion techniques, and outcome measures, complicating the capacity to derive conclusive conclusions regarding appropriate dose or delivery tactics [27]. These data collectively endorse the clinical advantages of MSCs, while underscoring the necessity for standardized procedures and extensive clinical studies to optimize the most effective parameters for MSC therapy in knee osteoarthritis.

Despite the growing evidence supporting the efficacy of intra-articular MSC therapy, the optimal dosing strategy remains unclear. Chen et al. [23] evaluated three doses of allogeneic AD-MSCs—16 million, 32 million, and 64 million cells—and observed that all groups achieved significant clinical improvement compared to control, with the 32 million group showing the most pronounced effect. However, the study did not perform or report direct statistical comparisons between doses [23]. As a result, although the 32M group showed numerically more significant improvement, the absence of head-to-head statistical testing limits conclusions regarding dose superiority. This highlights a recurring limitation in MSC trials, where dosing insights remain exploratory due to design or analytical constraints. Similarly, Sadri et al. [19] reported substantial clinical improvement following injection of 100 million AD-MSCs, but without comparison to lower doses, it remains uncertain whether such a high cell count offers added benefit [19]. In contrast, Gupta et al. [22] demonstrated significant improvements in WOMAC scores and cartilage preservation with just 25 million BM-MSCs, indicating that lower doses may be sufficient [22]. Matas et al. [24] compared single and repeated injections of 20 million UC-MSCs and reported greater efficacy with repeated dosing, suggesting that frequency and dose quantity may influence outcomes [24]. These variations in study design, cell source, and dosing strategy underscore the need for future head-to-head trials that systematically evaluate both the quantity and timing of MSC administration to determine the most effective and cost-efficient treatment protocols.

Several dose-escalation trials, excluded from the meta-analysis due to design limitations—such as the absence of patient-blinding or a control group—offer additional insights into the correlation between MSC dosage and therapeutic effects. Lamo-Espinosa et al. [28] examined 10 million and 100 million BM-MSCs, noting maintained clinical improvements in the high-dose group at 12 months,however, interpretation was constrained by the lower baseline OA severity in the low-dose cohort and the unblinded approach [28]. Lamo-Espinosa et al. [29] conducted a follow-up trial in which they gave 100 million BM-MSCs alongside PRGF, observing comparable enhancements to those achieved with PRGF alone, without any supplementary imaging advantages [29]. Matas et al. [30] performed a non-controlled, dose-escalation trial evaluating 2M, 20M, and 80M UC-MSCs. The trial revealed that lower doses yielded superior WOMAC improvements and safety outcomes, but all patients in the high-dose cohort encountered severe side effects [30]. These data underscore that increased MSC doses do not inherently enhance clinical outcomes and may even pose extra safety issues, emphasizing the necessity to establish an optimum and effective treatment window.

Moderate heterogeneity was observed in this meta-analysis (Q(6) = 11.95, *p* = 0.063; I2 = 49.8%; τ2 = 0.47), which is not unexpected given the variability in MSC sources, cell doses, and patient populations across studies. The use of a random-effects model appropriately accounted for this variation. Differences in the type of control used (saline, hyaluronic acid, or placebo), baseline disease severity, and delivery techniques may have contributed to between-study variance. However, future studies should aim for greater trial design and outcome reporting consistency to facilitate more precise comparisons and subgroup analyses.

The findings of this meta-analysis highlight the importance of dose efficiency in the clinical use of intra-articular MSC therapy. MSC doses of 25 million cells or less were associated with significant symptom improvement, while higher doses did not show added clinical benefit. This suggests that escalating cell quantity beyond a certain point may be unnecessary and economically inefficient, given cell-based therapies'high cost and complexity. Optimizing treatment with the minimum effective dose could improve cost-effectiveness, widen access, and simplify large-scale implementation. Notably, this raises the possibility that even lower doses could yield comparable outcomes. Future research should investigate this range to identify the optimal balance between therapeutic efficacy, safety, and affordability.

In addition to symptomatic improvement, several studies in this meta-analysis reported secondary outcomes that provide a broader view of MSC efficacy (Table 5). Imaging assessments using quantitative MRI techniques, such as T2 mapping and WORMS scoring, demonstrated improvements in cartilage quality or stabilization in MSC-treated patients compared to controls ([20–22]), supporting a potential disease-modifying effect. Similarly, quality-of-life outcomes based on SF-36 or SF-12 were reported in three studies. While Sadri et al. [19] observed significant improvements across all SF-36 domains, Vega et al. [20] and Matas et al. [24] reported no significant changes. These inconsistencies may reflect the limited sensitivity of generic QoL instruments in detecting changes in localized joint disease. Nevertheless, the addition of MRI and quality-of-life endpoints offers valuable insight into the structural and systemic impact of MSC therapy. It underscores the importance of including such outcomes in future trials.

**Table 5:** Secondary outcomes of the study

Study	Secondary Outcomes
[20]	VAS Pain: ↓ in MSC group (*p* = 0.01) MRI T2 mapping: Improved cartilage quality (*p* = 0.03) Lequesne Index & SF-12: Significant improvement in the MSC group Safety: No serious adverse events
[19]	VAS Pain: ↓ MSC vs placebo (*p* < 0.001) KOOS & SF-36: Significantly improved (*p* < 0.05) MRI: ↑ cartilage thickness medial tibia (*p* < 0.05–0.01) Immunology: ↑ IL-10, ↓ CD3/CD4/CD8, ↑ CD25 + (*p* < 0.005) Safety: Mild joint swelling, no serious AEs
[24]	VAS Pain: MSC-2: 2.4 ± 2.1 vs HA: 22.1 ± 9.8 (*p* = 0.03) MRI WORMS: No significant difference Safety: Mild effusion, no serious AEs
[22]	VAS and WOMAC subscales: all *p* < 0.001 MRI: No cartilage worsening in MSC group vs. worsening in placebo (*p* < 0.001) Cartilage volume: No significant change Safety: 5 mild/moderate AEs
[23]	VAS Pain: ↓ from 6.5 ± 1.6 to 2.0 ± 1.1 at 12 mo (*p* < 0.001) MRI: Cartilage improved in UC-MSC groups (A & C) Safety: Mild swelling in 2 cases, no severe AEs
[21]	MRI: Improved cartilage signal intensity Safety: One mild fever case, no serious AEs

This meta-analysis possesses multiple limitations. The quantity of included research was limited, and one study included numerous intervention arms, thereby heightening the possibility of unit-of-analysis bias. Despite employing a random-effects model to address inter-study variability, heterogeneity in MSC origin, dosage methodology, control type, and patient demographics may have impacted the aggregated values. This study focused solely on 12-month WOMAC scores due to their clinical significance and uniform reporting across trials. At the same time, other outcomes, including VAS or imaging evaluations such as MRI, were not examined. These secondary indicators may offer supplementary insights into the efficacy of MSCs. However, they are presently reported inconsistently in research. The meta-regression analysis on dose should be approached cautiously, as it was exploratory and constrained by the number of studies included. Subsequent trials should utilize standardized outcome measures, evaluate various dosage levels—particularly those under 25 million cells—and include long-term follow-up to determine the best treatment window and response durability accurately.

In evaluating the overall strength of these findings, the certainty of evidence was assessed using the GRADE framework. For the primary outcome of the WOMAC score at 12 months, the evidence was rated as low to moderate. This reflects the inclusion of randomized controlled trials with generally low risk of bias, but with moderate heterogeneity and some imprecision due to small sample sizes and wide confidence intervals. No serious indirectness was identified, as the studies directly addressed the population, intervention, and outcomes of interest. Although publication bias could not be thoroughly assessed due to the limited number of studies, the consistency of effect direction across trials adds credibility. Despite these limitations, the findings offer meaningful guidance for clinical application and future research: low-dose MSC therapy may be sufficient, more cost-effective, and potentially safer, without compromising efficacy. High-quality, head-to-head trials comparing different MSC doses are needed to confirm these conclusions and to establish an optimal dosing window.

## Conclusion

This meta-analysis demonstrates that intra-articular MSC therapy provides significant clinical benefit in patients with knee osteoarthritis, with moderate-to-large effect sizes observed in WOMAC score improvements at 12-month follow-up. The findings confirm the therapeutic potential of MSCs for symptom relief and functional improvement. Notably, 25 million cells or fewer doses were associated with statistically significant improvements, while higher doses did not yield superior outcomes. This suggests a lower MSC dose may be sufficient, supporting the case for dose-efficient treatment strategies.

While promising, the evidence base remains limited by heterogeneity in MSC sources, study protocols, and outcome measures. Additionally, several dose-escalation studies not included in the meta-analysis due to methodological limitations further highlight the complexity of dose–response relationships and suggest that excessively high doses may not enhance efficacy and could compromise safety. Future well-controlled, head-to-head trials are needed to evaluate lower-dose regimens and establish optimal dosing windows. Standardization of protocols and long-term follow-up will be essential to confirm the durability of response and to guide the integration of MSC therapy into clinical practice.

## Data Availability

No datasets were generated or analysed during the current study.
